# Clinical features of gender self-identification disorder arising within non-psychotic forms of endogenous illnesses and personality disorders

**DOI:** 10.1192/j.eurpsy.2026.11925

**Published:** 2026-07-31

**Authors:** S. Sorokin, G. Popova, K. Kostenkova

**Affiliations:** 1 Department for the Study of Endogenous Mental Disorders and Affective States; 2 Department of Early Childhood Psychiatry, Mental health research center; 3 Psychiatric Hospital no. 1 Named after N.A. Alexeev, Moscow, Russian Federation

## Abstract

**Introduction:**

The recent rise in consultations for gender dysphoria (GD) is largely driven by exogenous factors: globalization, virtualization of social life, ubiquitous and diverse information flow, intensified cross-group communication and virtual communities, easy access to medical content, and a cultural shift that romanticizes mental illness.

**Objectives:**

To determine clinical features of gender identity disorder (GID) arising within endogenous non-psychotic illnesses and personality disorders, and to identify patterns of course and outcomes.

**Methods:**

63 patients (46 women, 17 men; mean age 19.4 years) with GID formed within a depressive state in the context of schizotypal disorder, histrionic personality disorder, or hystero-schizoid spectrum personality disorders.

**Results:**

**Schizotypal disorder** (50.8%): GID appeared during depressive episodes of varying severity; after depression remitted, its intensity did not consistently track depressive symptoms. Manifestations included eccentric, idiosyncratic attempts to approximate the opposite sex, applications to change legal gender marker, and unsupervised HRT. Symptoms largely persisted despite treatment, with only partial reductions/transformation in some; social adaptation declined over 4 years. **Hystero-schizoid spectrum PDs** (30.1%): GID emerged during depressive decompensation (usually moderate), often outlasting the depressive phase and persisting in compensation. Depth was substantial: external changes, initiation of HRT, plans for surgery; by follow-up, no surgeries occurred. Two patients achieved full remission. Social adaptation remained relatively high. **Histrionic PD** (19.0%): GID arose with mild–moderate depression, did not outlast the period of maladaptation, was non-persistent, led to no surgery, and in some cases fully remitted. Social adaptation stayed high.

**Conclusions:**

Identifying the underlying disorder within which GID presents helps estimate its likely course and outcomes.

**Disclosure of Interest:**

None Declared

